# The wild side of plant microbiomes

**DOI:** 10.1186/s40168-018-0519-z

**Published:** 2018-08-16

**Authors:** Juan E. Pérez-Jaramillo, Víctor J. Carrión, Mattias de Hollander, Jos M. Raaijmakers

**Affiliations:** 1 Department of Microbial Ecology, Netherlands Institute of Ecology (NIOO-KNAW), P.O. Box 50, 6708 PB Wageningen, The Netherlands; 2 Institute of Biology, Leiden University, Sylviusweg 72, 2333 BE Leiden, The Netherlands

Plants rely on their microbiome for a number of life-support functions including nutrient acquisition and protection against (a)biotic stress factors. For crop plants, however, the process of domestication may have adversely impacted the composition and functions of the associated microbiota, thereby undermining their beneficial effects on plant growth and health. Here, we conducted a meta-analysis to resolve if and how plant domestication affected the composition of the root-associated microbiome. For different plant species, we observed significant and consistent differences in the abundance of Bacteroidetes, Actinobacteria, and Proteobacteria. Potential causes and consequences of these microbiome shifts following plant domestication are discussed.

In the search for new strategies to engineer “healthy microbiomes” of plants and humans, considerable attention is given to coevolutionary signatures of host-microbe interactions and mechanisms involved in microbiome assembly and activity [1–3]. For example, comparative analyses of the human microbiome revealed a higher abundance of Bacteroidetes in the gut of hunter-gatherer populations of rural communities in non-industrialized regions than in the gut of Westernized populations, a distinct divergence that appears to be associated with differences in the content of starch, fiber, and plant polysaccharides in the food [4, 5]. Similarly, shifts in the gut microbiome composition in captive mammals as compared to their wild counterparts have been associated with a loss of dietary fiber and a potential increase in protein consumption [6, 7]. Interestingly, one of the most relevant changes in the gut microbiome of mammals in captivity is an increase in the relative abundance of the genus *Bacteroides* and a decrease of the genus *Prevotella*, both from the Bacteroidetes phylum, a pattern that has also been observed in Westernized humans [6]. For plants, several studies have suggested that domestication altered the composition of the root microbiome with an adverse effect on the association with symbiotic nitrogen-fixing rhizobia and mycorrhizal fungi [8]. For instance, Kiers et al. showed that older soybean cultivars had a higher yield difference ratio, i.e., the ability of soybean cultivars to reach their full symbiotic potential in the presence of a mix of rhizobial strains with different symbiotic effectiveness, as compared to newer soybean cultivars [9]. Similarly, it has been shown that wild ancestors and primitive landraces of wheat, breadfruit, and maize can benefit more from mycorrhizal symbiosis than modern cultivars [10–13]. To date, however, the impact of plant domestication on the vast majority of other root-associated microorganisms is not well understood. In a recent study, we revealed that the rhizosphere microbiome of wild relatives of common bean (*Phaseolus vulgaris*) harbored a higher abundance of Bacteroidetes, while the root microbiome of modern bean accessions was dominated by bacterial families belonging to the Actinobacteria and Proteobacteria [14]. Also, studies on other plants species, including *Arabidopsis* [15], sugar beet [16], barley [17], and lettuce [18], suggested that domestication led to compositional changes in the root microbiome. To investigate if these effects of domestication cause similar shifts in microbiome composition for multiple plant species, we set out a meta-analysis of the root microbiome of various crop plants and their wild relatives. The specific objectives of this computational “walk on the wild side” were to (i) determine the differences and patterns in root microbiome composition between wild relatives and their domesticated counterparts and (ii) identify the relative abundance of specific taxa within the Bacteroidetes phylum for crop plants and their wild relatives. To this end, we retrieved the raw 16S rDNA sequences from six independent common garden experiments with a total of nine plant species and adopted the same computational pipeline to assess the root/rhizosphere bacterial community composition (Additional file 1: Table S1, Additional file 2: Table S2, and Additional file 3). Regarding the analysis of the *Arabidopsis* root microbiome by Schlaeppi et al., our comparison was made based on the divergence time estimates with *Cardamine hirsuta* considered as the “ancient/wild” species and members of the genus *Arabidopsis* as the “modern/evolved” counterpart.

First, we observed marked differences in the diversity of bacterial communities associated with roots of the different plant species, which were largely explained by the study (29.1%, PERMANOVA, *P* < 0.001) (Additional file 4: Figure S1) and the microhabitat sampled, i.e., root or rhizosphere (Additional file 4: Figure S2). These results reinforce the preponderant role of soil type in the assembly of the root microbiome [19]. Also, the higher diversity in the rhizosphere as compared to the endosphere (Additional file 4: Figure S2) is in accordance with previous reports [20]. Subsequent pairwise comparisons showed that, for each plant species, the Bacteroidetes were consistently enriched in the root or rhizosphere of the wild relatives, and a comparable difference was observed between *Cardamine hirsuta* and *Arabidopsis halleri* (moderated *t* tests; *P* < 0.05, BH corrected) (Fig. 1a). For the ancestor of sugar beet, *Beta vulgaris* ssp. *maritima*, we also observed a higher prevalence of Bacteroidetes taxa as compared to modern sugar beet, although this difference could not be analyzed statistically as the replicate samples in that study [16] were pooled. Next to the Bacteroidetes, we observed a higher relative abundance of some other bacterial families on the roots of wild relatives of the different plant species. In common bean, Planctomycetes, Verrucomicrobia, and Acidobacteria together with some Proteobacteria families were also more abundant on the roots of the wild accession. For wild barley, a few Proteobacteria families were enriched as well as two Firmicutes families. For wild lettuce and *Cardamine hirsuta*, also several Proteobacteria families were enriched. Overall, Proteobacteria and Actinobacteria were consistently enriched on the roots of the modern counterpart, while Bacteroidetes was found almost exclusively enriched on the roots of the wild relatives irrespective of the plant species and study. The phylum Bacteroidetes has also been found as a prevalent and abundant member in the rhizosphere of several other wild plant species [21, 22].

**Fig. 1:**
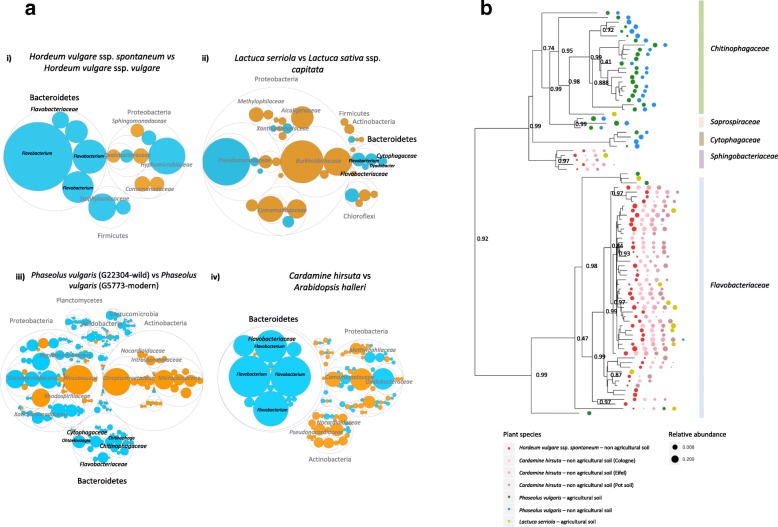
Enrichment and taxonomic diversity of bacterial taxa in wild and domesticated plant species. **a** Differential abundance of bacterial OTUs between wild plant accessions and their domesticated counterparts. Presented here are selected pairwise comparisons between (i) wild barley (*Hordeum vulgare* ssp. *spontaneum*) and modern barley (*Hordeum vulgare* ssp. *vulgare*), (ii) wild lettuce (*Lactuca serriola*) and cultivated lettuce (*Lactuca sativa* ssp. *capitata*), (iii) wild and modern accessions of common bean (*Phaseolus vulgaris*), and (iv) *Cardamine hirsuta* and *Arabidopsis halleri*. Each comparison was made using a zero-inflated Gaussian distribution mixture model followed by moderated *t* test and a Bayesian approach. Only OTUs significantly enriched in one of the two accessions are shown (FDR < 0.05). The largest circles represent the phylum level, and the inner circles represent the class and family level. The color of the circles represents the OTUs enriched in the rhizosphere/roots of wild relatives (cyan) or of modern crop plants (orange), with the assigned genus in italics. The size of the circle is the mean read relative abundance of the differentially abundant OTU. **b** Phylogenetic tree of bacterial members of the Bacteroidetes phylum associated with different wild plant species. The Bacteroidetes taxa were selected from microbiome data of wild plant species to construct the phylogenetic tree. The size of the circles corresponds to the relative abundance for each Bacteroidetes taxa. Only the data with a relative abundance higher than 0.1% is depicted in the tree. Each abundance data is the average of at least three samples per plant species and site

Our analysis further revealed that the extent of the Bacteroidetes enrichment on the roots of wild plant relatives exhibits plant species-specific signatures. For example, approximately 50% of the bacterial species differentially enriched on the roots of wild barley belonged to the Bacteroidetes, while for *Cardamine hirsuta*, wild lettuce, and wild common bean, the Bacteroidetes represented 33.3, 24.5, and 18.9%, respectively, of the root-associated bacterial community. Subsequent phylogenetic analysis of the Bacteroidetes that were more abundant (> 0.1%) on the wild relatives showed two main clusters: one composed mainly of the members of the *Chitinophagaceae* family and the other of the members of the *Flavobacteriaceae* family (Fig. 1b). The family *Flavobacteriaceae* was represented by a high diversity in *Cardamine hirsuta* and wild barley, whereas *Chitinophagaceae* and *Cytophagaceae* families were predominant in the root microbiome of wild relatives of common bean (Fig. 1b). Collectively, these results indicate that plant domestication resulted in a similar overall taxonomic shift in the prokaryotic root microbiome with a reduced abundance of the Bacteroidetes phylum on modern accessions and a concomitant increase of members of the Actinobacteria and Proteobacteria (Fig. 2). At higher taxonomic levels, we observed that the plant species-specific effects observed on Bacteroidetes families may be probably due to the differences in the physicochemical characteristics of the diverse soils used in these independent studies, such as divergent pH values and the organic carbon content (Additional file 1: Table S1).

**Fig. 2:**
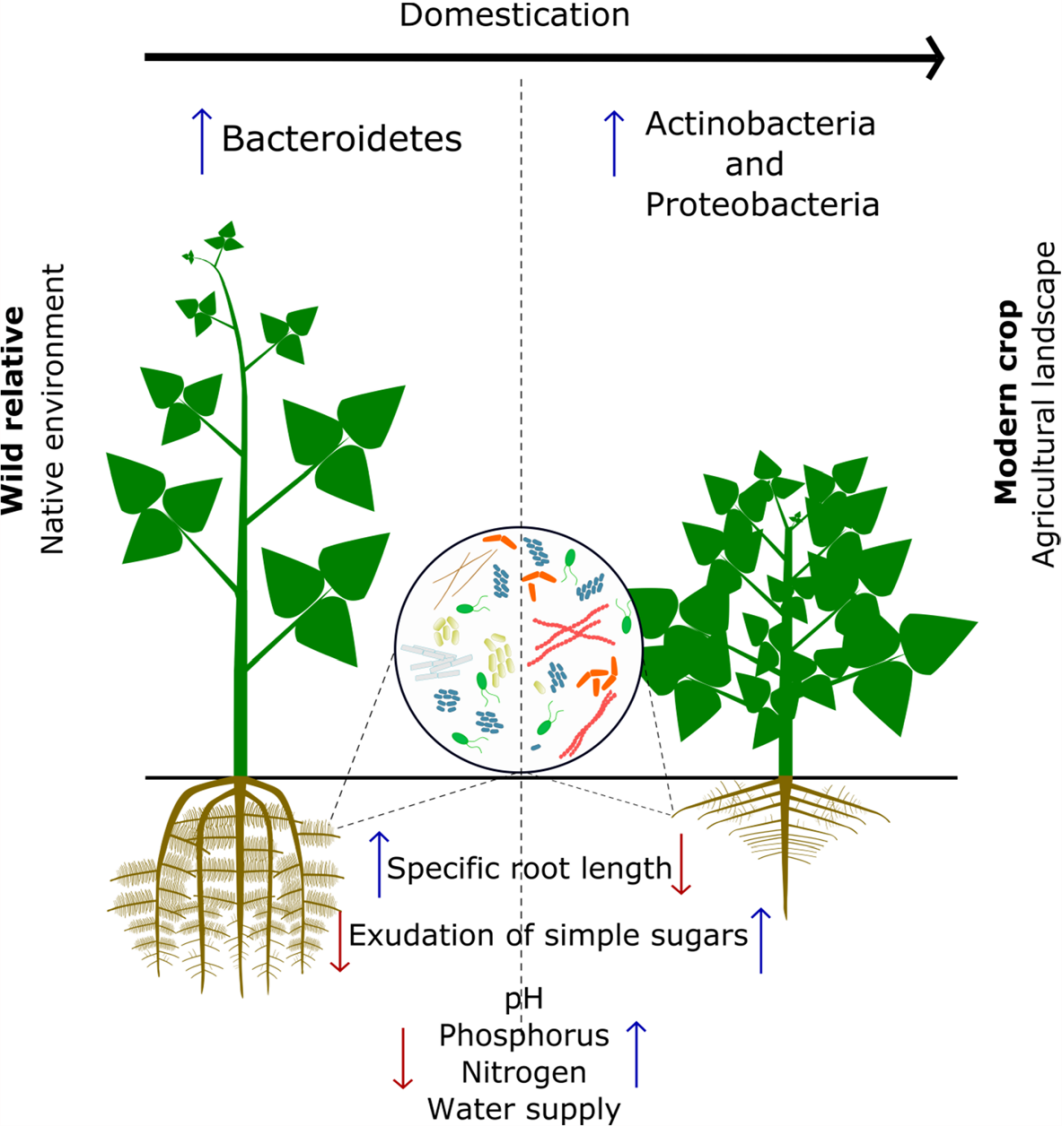
Impact of domestication on soil management, plant phenotype, plant physiology, and rhizobacterial diversity. In this hypothetical schematic representation, the root morphology of the wild relative substantially differs from that of the modern counterpart. Readily available macronutrients and water associated with agricultural management led to shallower roots in the modern crop cultivars as compared to the roots of the wild relatives, which are rooting deeper with conspicuous lateral roots. Domesticated crop plants presumably also exude more “simple” sugars than their wild relatives. The impact of the domestication process on rhizobacterial community composition is reflected in a decrease in Bacteroidetes abundance on modern crop plants, while the abundances of the Actinobacteria and Proteobacteria are increased

Firstly, it is important to emphasize that in our analyses, the same computational pipeline was used adopting a rarefaction of the operational taxonomic unit (OTU) table to the same sequencing depth. However, the approach used in this study cannot address all biases associated with this type of meta-analysis. Differences in soil types, sampling strategies, nucleic acid extraction protocols, and sequencing techniques between the different studies may have affected the reach of our meta-analysis and the interpretation of the results. Nevertheless, it is noteworthy that despite all these constraints, we found similar and consistent differences between the prokaryotic composition of the root/rhizosphere microbiome of wild and domesticated plant species with a significantly higher abundance of Bacteroidetes on/in the roots of wild plant relatives. Why Bacteroidetes are relatively more abundant in the root and rhizosphere compartments of wild relatives of various crop plant species is yet unknown. They are recognized for their ability to degrade complex biopolymers, a trait associated with a diverse set of carbohydrate processing enzymes [23, 24]. Hence, their prevalence in the root compartments of wild plant species may be a phylogenetic signal associated with the presence of complex biopolymers in their root exudates (Fig. 2). Plant root exudates can have a major impact on the structure and functioning of microbial communities in soil environments [25, 26]. A recent study on mutants of poplar trees, silenced in the cinnamyl-Co reductase (*CCR*) gene of the monolignol-specific lignin pathways, showed significant effects on the density and composition of culturable rhizosphere and endosphere bacteria, microbiome shifts that were proposed to be mediated, at least in part, by changes in extractable plant phenolic compounds such as ferulic acid [27]. In this context, it is worth noting that one of the most common domestication syndrome traits is related with the changes in the type and amount of secondary metabolites, such as the loss of specific compounds that are toxic for humans or livestock or the reduction of flavonoid content in the leaves [28–30]. To date, however, very little is known about the qualitative and quantitative differences between root exudation profiles of crop plants and their wild relatives. For wheat, it has been shown that a modern wheat variety exuded three to five times more “simple” sugars (mainly fructose, glucose, and maltose) than an ancient wheat cultivar under stress conditions, a feature that might be related with a lower capacity of the modern wheat cultivar to control sugar exudation [31]. Whether the higher levels of these “easy-digestible” sugars are also the case for other plant species and may contribute to a competitive advantage and a concomitant higher abundance of Proteobacteria and Actinobacteria on the roots of modern crop cultivars remain to be addressed.

Also, differences in root architecture between crop plants and their wild relatives may impact root microbiome assembly. More specifically, the prevalence of Bacteroidetes in the rhizosphere of wild bean correlated significantly with a higher specific root length (SRL, i.e., root length per unit of root dry mass) and a lower root density [14]. A high SRL has been associated with a higher efficiency of water search and uptake for the plant and is considered a strategy to acquire nutrients in low-fertile soils [32, 33]. Along with the changes in plant genotype and phenotype, the domestication process also involves changes in the environment and the concomitant need of management practices, such as the use of chemical pesticides and fertilizers, to sustain growth and health of the crop plants [8]. Therefore, altered root morphology traits (Fig. 2) as well as changes in plant physiology and root exudation may have contributed to the observed and consistent shifts in the prokaryotic root microbiomes between wild plant relatives and their domesticated counterpart. This hypothesis needs to be validated by experiments where morphological and physiological traits, in particular, root architecture and exudation profiles, of wild relatives of crop plants are assessed in agricultural soils as well as in soils from their centers of origin and diversification.

Whether a higher relative abundance of Bacteroidetes affects plant growth and health as was shown for growth (i.e., obesity) and health of humans [34–36] is not known to date. Some studies suggested that representatives of this phylum can affect plant growth and health. In particular, strains of the genus *Flavobacterium* have been associated with plant growth promotion and disease protection [37]. For the legume plant *Trifolium pratense*, however, *Flavobacterium* led to impaired shoot growth [38]. For the genus *Chryseobacterium*, disease protective effects have been described [39], but effects on plant growth and health by most other Bacteroidetes, including members of the *Chitinophagaceae* and *Cytophagaceae* families detected here, remain to be discovered. Establishing a phenotypically and genomically diverse and well-characterized collection of Bacteroidetes species from multiple wild plant relatives followed by controlled bioassays to test the effects of individual species/strains and consortia on plant growth and health under diverse environmental conditions will shed more light on their functional importance for the growth and survival of wild plant species in their native, environmentally harsh habitats. Understanding the functional importance of these “missing plant microbes” can be highly instrumental in plant breeding programs and for improving our future crop production systems in a changing environment.

## Methods

All methods are described in detail in the Additional file 3.

## Additional files

Additional file 1: **Table S1**. General information of the datasets used for the meta-analysis. (PDF 466 kb)

Additional file 2: **Table S2**. Physicochemical characteristics of the soils used in the studies used in the meta-analysis. (PDF 443 kb)

Additional file 3: Methods section. (PDF 402 kb)

Additional file 4: **Figure S1**. Rhizosphere bacterial community composition across studies of wild, landrace, and modern plants. **Figure S2**. α-diversity of 16S sequence data of wild, landrace, and modern plant species for rhizosphere and roots. (PDF 853 kb)
