# Supplementary material for: The wild side of plant microbiomes
Source: Microbiome. 2018 Aug 16;6:143. doi: 10.1186/s40168-018-0519-z (PMC6097318; doi:10.1186/s40168-018-0519-z)
Supplement: Supplementary file 1 — Table S1. General information of the datasets used for the meta-analysis. (PDF 466 kb) [file 40168_2018_519_MOESM1_ESM.pdf]

Supplementary Table S1. General information of the datasets used for the meta-analysis.

Study	Sequencing Method	Repository	Project code	16S primer pair	Plant species and subspecies	Domestication status	DOI
Zachow *et al.*, 2014	454	NCBI*	PRJNA233435	V4-V5	*Beta vulgaris* ssp. *vulgaris*	Domesticated beet	10.3389/fmicb.2014.00415
					Beta vulgaris ssp. *maritima*	Wild relative	
Schlaeppi *et al.*, 2014	454	ENA**	PRJEB5058	V5-V6-V7	*Cardamine hirsuta*	Wild plant	10.1073/pnas.1321597111
					Arabidopsis halleri	Wild plant	
					Arabidopsis lyrata	Wild plant	
					Arabidopsis thaliana	Wild plant	
Bulgarelli *et al.*, 2015	454	ENA	PRJEB5860	V5-V6-V7	*Hordeum vulgare* ssp. *spontaneum*	Wild relative	10.1016/j.chom.2015.01.011
					Hordeum vulgare ssp. *vulgare*	Domesticated barley	
Cardinale *et al.*, 2015	454	ENA	PRJEB5101	V4	*Lactuca serriola*	Wild relative	10.1111/1462-2920.12686
					Lactuca sativa ssp. *capitata*	Domesticated lettuce	
					Lactuca sativa ssp. *crispa*	Domesticated lettuce	
					Lactuca sativa ssp. *longifolia*	Domesticated lettuce	
					Lactuca sativa ssp. *augustana*	Domesticated lettuce	
Leff *et al.*, 2017	MiSeq	NCBI	SRP075934	V4	*Helianthus annuus*	Wild, landraces and modern accessions included ***	10.1111/nph.14323
Pérez-Jaramillo *et al.*, 2017	MiSeq	ENA	PRJEB19467	V3-V4	*Phaseolus vulgaris*	Wild, landraces and modern accessions included ***	10.1038/ismej.2017.85

*National Center for Biotechnology Information of the United States of America

**European Nucleotide Archive

***Wild; plants that have a ready ability to grow freely in natural ecosystems, with strong dispersal mechanisms; Landrace: locally developed crop varieties by farmers within their own agricultural, horticultural or agri-silvicultural systems; Modern accessions or modern improved varieties are the result of plant breeding in the pursue of higher yields, better quality and more stable production, typically grown on heavily managed agricultural settings.
