# Supplementary material for: The wild side of plant microbiomes
Source: Microbiome. 2018 Aug 16;6:143. doi: 10.1186/s40168-018-0519-z (PMC6097318; doi:10.1186/s40168-018-0519-z)
Supplement: Supplementary file 2 — Table S2. Physicochemical characteristics of the soils used in the studies used in the meta-analysis. (PDF 443 kb) [file 40168_2018_519_MOESM2_ESM.pdf]

Supplementary Table S2. Physicochemical characteristics of the soils used in the studies used in the meta-analysis.

Study	Soil type used for plant growth						Type of experiment
	Texture (%)			Classification	pH	Organic C (%)	
	Clay	Silt	Sand				
Zachow *et al.,* 2014	NA*	NA	NA	Clay	9.5	NA	Field/Pot
Schlaeppi *et al.*, 2014	13.4	37.3	49.3	Sandy Loam	6.95	4.0	Pot trial
Bulgarelli *et al.,* 2015	4.2	4.2	91.6	Sand	7.12	1.0	Pot trial
Cardinale *et al.,* 2015	NA	NA	NA	NA	NA	NA	Field
Leff *et al.,* 2017	NA	NA	NA	Sandy Loam	NA	NA	Field
Pérez-Jaramillo *et al.,* 2017	8	30	62	Clay Loam	5.8	17.9**	Pot trial

*Not available

*Organic Matter
