# Supplementary material for: The wild side of plant microbiomes
Source: Microbiome. 2018 Aug 16;6:143. doi: 10.1186/s40168-018-0519-z (PMC6097318; doi:10.1186/s40168-018-0519-z)
Supplement: Supplementary file 3 — Methods section. (PDF 402 kb) [file 40168_2018_519_MOESM3_ESM.pdf]

The wild side of plant microbiomes

Juan E. Pérez-Jaramillo, Victor J. Carrión, Mattias de Hollander and Jos M. Raaijmakers

Supplementary Material

Methods

Processing of the sequences

Per sample fastq files of 16S metagenome amplicon sequences were kindly provided by the authors of the different studies [1, 6]. The reads were quality filtered for single end reads with sickle [7], and bases below phred score 36 and shorter than 100bp were trimmed. Only high quality filtered reads were mapped to full length 16S sequences from the Silva 119 release [8] using the usearch global algorithm implemented in VSEARCH version 1.9.6 [9]. The alignment results were directly converted to BIOM format using biom version 2.1.5 [10]. Consensus/majority taxonomy was added as metadata to the biom file. Finally, all BIOM files of each dataset were merged using Qiime version 1.9.1 [11]. The Silva 119 reference phylogenetic tree provided by Qiime (clustered at 97%) was filtered using the Qiime command *filter_tree.py* to keep Bacteroidetes taxa which were present only in wild plants. Subsequently, we built phylogenetic trees using the Phyloseq package in R, and for graphic purposes only branches with a relative abundance higher than 0.1% from the total amount of reads were kept.

Description of plant and soils used in the studies

In the study by Pérez-Jaramillo *et al.*, 2017 [6], the wild and modern common bean accessions (*Phaseolus vulgaris*) were cultivated in the same soil in a pot trial under the same climatic conditions followed by characterization of the rhizosphere microbiome composition. For

Cardinale *et al.*, 2015 [4], it is described in the main text that the experiments with *Lactuca serriola*, wild relative of lettuce, and four subspecies of *L. sativa* were done in the field in an experimental farm in Austria, followed by characterization of the rhizosphere microbiome composition. Nevertheless, the description of soil characteristics is not provided and therefore it is not possible to describe growth conditions for the plants neither. In the study by Zachow *et al.*, 2014 [2], wild beet plants were collected in the drift line at the Mediterranean Sea coast in Slovenia. From the same region, soil from the coastal drift line was collected and used under unspecified greenhouse conditions in order to grow domesticated beet in the same soil than wild beet. For the study by Schlaeppi *et al.*, 2014 [1], several field experiments and greenhouse experiments were done and four different types of soil were used. The root microbiome composition was characterized for *Arabidopsis thaliana*, *Cardamine hirsuta*, *A. halleri* and *A. lyrata*, while the rhizosphere microbiome composition was characterized for *A. thaliana* and *C. hirsuta*. The latter is an *Arabidopsis* relative species which diverged ~35 Mya and is phylogenetically the most distant species. Finally, in the study by Bulgarelli *et al.*, 2015 [3], the microbiome composition of root and rhizosphere compartments of wild barley (*Hordeum vulgare* spp. *spontaneum*), a landrace and a modern variety of barley (*H. vulgare* spp. *vulgare*) were characterized. For this, two pot trials were performed with the three plant accessions in soils that were collected in the same location in two different years. All the information about plant accessions, soil type and experimental conditions are described in the supplementary tables S1 and S2.

Statistical analysis

In order to compare the different datasets we rarefied the OTU table up to 500 reads, which was the sequencing depth that allows us to work with most of the data sets available. All the data sets were included except for the data of Leff *et al.*, 2017, for which sequencing depth,

after processing with the method described above, did not reach the threshold implemented. For Alpha diversity metrics, the command *alpha_diversity.py* in Qiime was applied and the output files were retrieved and plotted in R using the package ggplot2 (v.2.0.0) [12]. As we did not observe significant differences in alpha diversity indexes between wild and domesticated accessions of the same plant species, the data was merged per plant species in order to illustrate exclusively differences between compartments (root/rhizosphere). For beta-diversity calculations, a Bray–Curtis dissimilarity matrix was calculated and used it to build Principal Coordinate Analyses and Permutational multivariate analyses of variance (Adonis function) were performed to evaluate the significance of the variables tested, both retrieved from Phyloseq (v.1.10) [13] and Vegan (v.2.4-4) [14]. For the OTU level analysis, the function calculateEffectiveSamples from the metagenomeSeq R package (v.1.12) [15] was applied to the filtered OTU table and features with less than the average number of effective samples in all features were removed. For the analysis at OTU level, we used normalized tables applying a cumulative-sum scaling normalization. Then, a Zero-Inflated Gaussian Distribution Mixture Model was applied using the fitZig function from metagenomeSeq. With the coefficients from the model, we applied moderated t-tests between accessions using the makeContrasts and eBayes commands retrieved from the R package Limma (v.3.22.7) [16]. Obtained P-values were adjusted using the Benjamini–Hochberg correction method. Differences in the abundance of taxa between accessions were considered significant when adjusted P-values were lower than 0.05 at OTU level. Treemap (v.3.7.3) was used to visualize the significantly abundant OTU’s, the taxonomy, the adjusted *P*-value and per mil relative abundance in bubble graphs, in which the size of the bubbles indicates the relative abundance per hundred of the raw read counts.

References

1. Schlaeppi K, Dombrowski N, Oter RG, Ver Loren van Themaat E, Schulze-Lefert P. Quantitative divergence of the bacterial root microbiota in *Arabidopsis thaliana* relatives. Proc Natl Acad Sci. 2014;111:585–92.

2. Zachow C, Müller H, Tilcher R, Berg G. Differences between the rhizosphere microbiome of *Beta vulgaris* ssp. *maritima*-ancestor of all beet crops-and modern sugar beets. Front Microbiol. 2014;5:415.

3. Bulgarelli D, Garrido-Oter R, Münch PC, Weiman A, Dröge J, Pan Y, et al. Structure and function of the bacterial root microbiota in wild and domesticated barley. Cell Host Microbe. 2015;17:392–403.

4. Cardinale M, Grube M, Erlacher A, Quehenberger J, Berg G. Bacterial networks and co-occurrence relationships in the lettuce root microbiota. Environ Microbiol. 2015;17:239-52.

5. Leff JW, Lynch RC, Kane NC, Fierer N. Plant domestication and the assembly of bacterial and fungal communities associated with strains of the common sunflower, *Helianthus annuus*. New Phytol. 2017;214:412-23.

6. Pérez-Jaramillo JE, Carrión VJ, Bosse M, Ferrão LFV, de Hollander M, Garcia AAF, et al. Linking rhizosphere microbiome composition of wild and domesticated *Phaseolus vulgaris* to genotypic and root phenotypic traits. ISME J. 2017;11:2244–57.

7. Joshi NA, Fass JN. Sickle: A sliding-window, adaptive, quality-based trimming tool for FastQ files. (Version 1.33) [Software]. 2011. Available at https://github.com/najoshi/sickle.

8. Quast C, Pruesse E, Yilmaz P, Gerken J, Schweer T, Yarza P, et al. The SILVA ribosomal RNA gene database project: improved data processing and web-based tools. Nucleic Acids Res. 2013;41:D590–D596.

9. Rognes T, Flouri T, Nichols B, Quince C, Mahé F. VSEARCH: a versatile open source tool for metagenomics. PeerJ. 2016;4:e2584.

10. McDonald D, Clemente JC, Kuczynski J, Rideout JR, Stombaugh J, Wendel D, et al. The Biological Observation Matrix (BIOM) format or: how I learned to stop worrying and love the ome-ome. Gigascience. 2012;1:7.

11. Caporaso JG, Kuczynski J, Stombaugh J, Bittinger K, Bushman FD, Costello EK, et al. QIIME allows analysis of high-throughput community sequencing data. Nat Methods. 2010;7:335–336.

12. Wickham, H. ggplot2: elegant graphics for data analysis. New York: Springer-Verlag; 2009

13. McMurdie PJ, Holmes S. phyloseq: An R package for reproducible interactive analysis and graphics of microbiome census data. PLOS ONE. 2013;8:e61217.

14. Oksanen J, Kindt R, Legendre P, O'Hara B. vegan: Community Ecology Package. R package version 2.4-4. http://CRAN.R-project.org/package=vegan; 2017.

15. Paulson JN, Talukder H, Pop M, Bravo HC. metagenomeSeq: Statistical analysis for sparse high-throughput sequencing. R package version 1.16.0. http://cbcb.umd.edu/software/metagenomeSeq; 2017.

16. Ritchie ME, Phipson B, Wu D, Hu Y, Law CW, Shi W, et al. limma powers differential expression analyses for RNA-sequencing and microarray studies. Nucleic Acids Res. 2015;43:e47.
