# Supplementary material for: The wild side of plant microbiomes
Source: Microbiome. 2018 Aug 16;6:143. doi: 10.1186/s40168-018-0519-z (PMC6097318; doi:10.1186/s40168-018-0519-z)
Supplement: Supplementary file 4 — Figure S1. Rhizosphere bacterial community composition across studies of wild, landrace, and modern plants. Figure S2. α-diversity of 16S sequence data of wild, landrace, and modern plant species for rhizosphere and roots. (PDF 853 kb) [file 40168_2018_519_MOESM4_ESM.pdf]

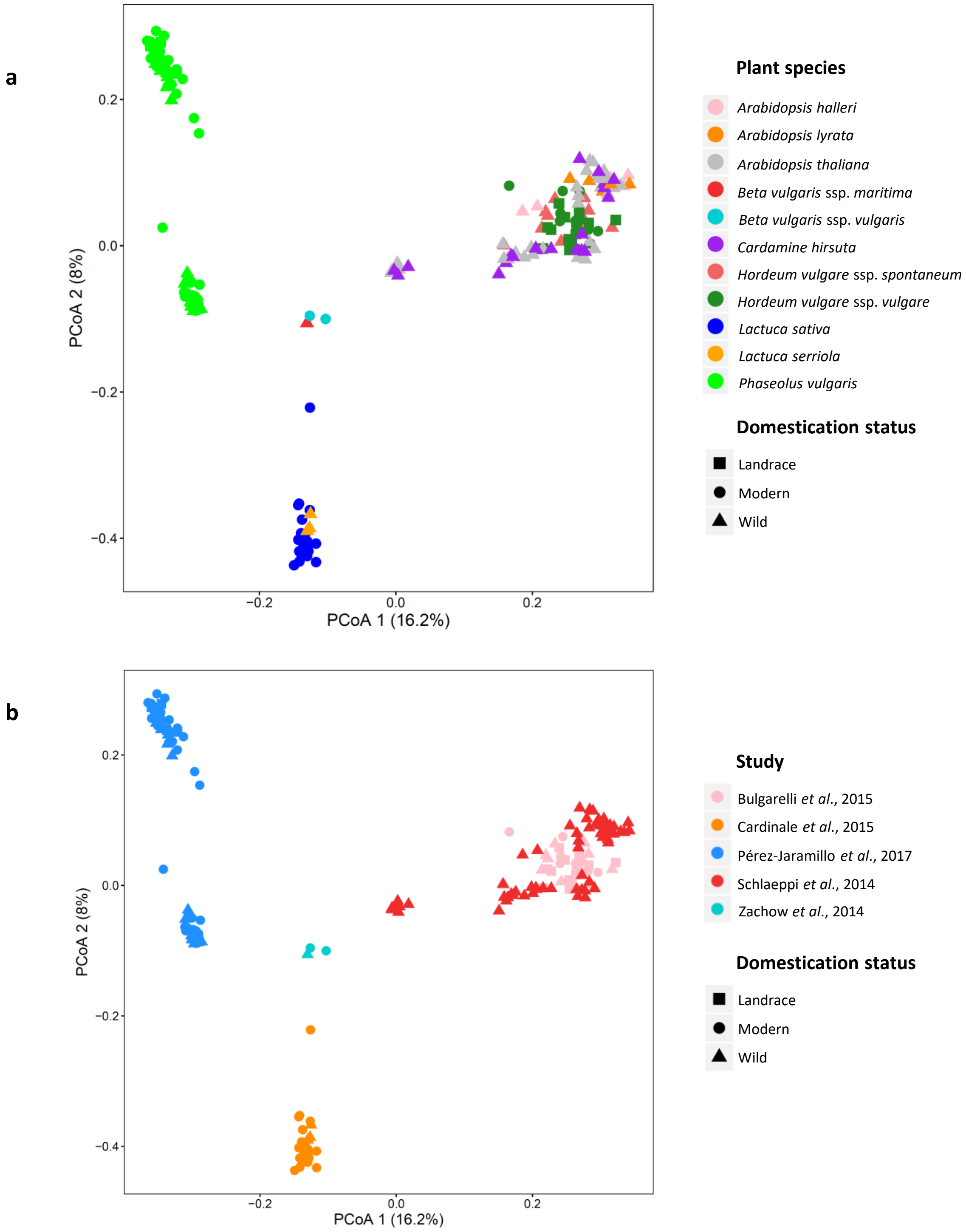

Supplementary Figure 1. Rhizosphere bacterial community composition across studies of wild, landrace and modern plants. Principal Coordinate Analysis (PCoA) of Bray-Curtis dissimilarities of 16S rRNA data. **a)** PCoA with samples colored by plant species. b) PCoA with samples colored by study. The source of the data (study) was the main explaining variable as assessed by PERMANOVA (29.1%; P<0.005).

a

b

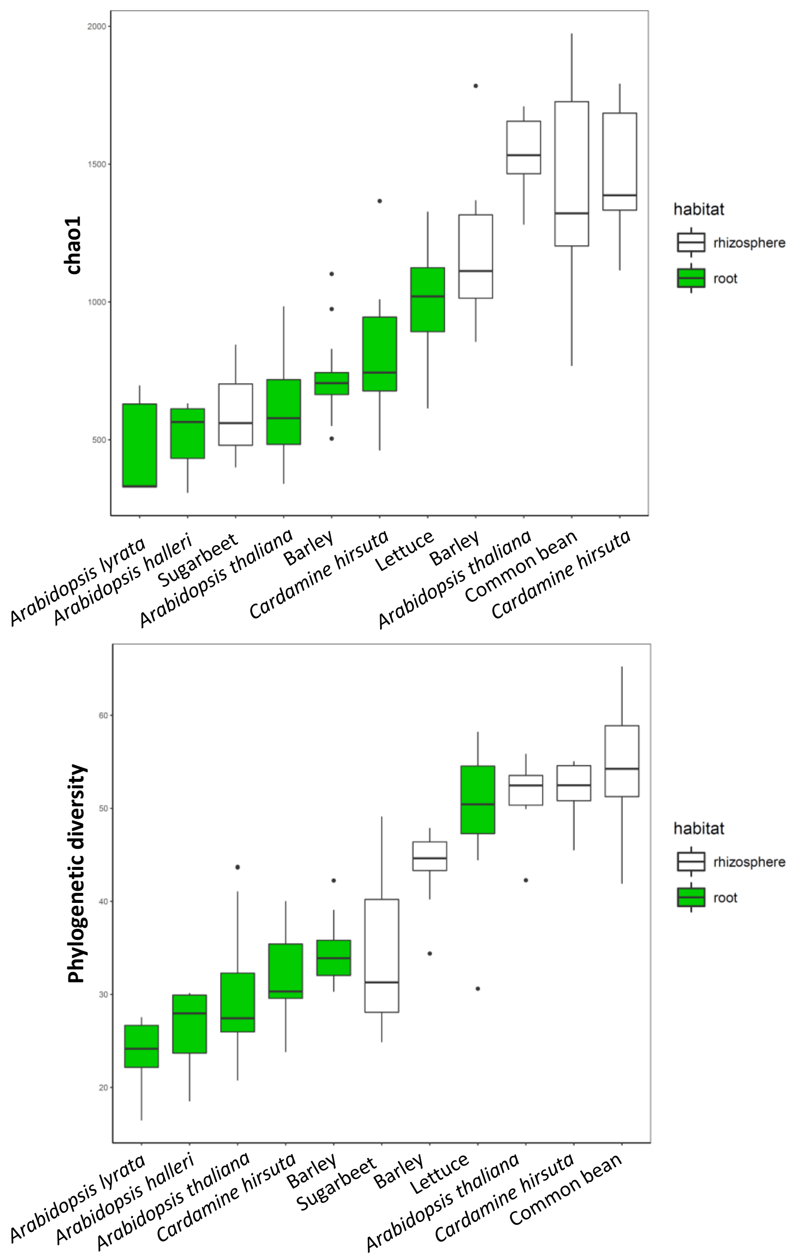

Supplementary Figure 2. α-diversity of 16S sequence data of wild, landrace and modern plant species for rhizosphere and roots. (a) Chao1 and **(b)** Phylogenetic diversity for all the plants included in the meta-analysis. Bacterial diversity on/in the roots is less than in the rhizosphere.
